# Supplementary material for: The interactome of intact mitochondria by cross-linking mass spectrometry provides evidence for coexisting respiratory supercomplexes
Source: Mol Cell Proteomics. 2017 Dec 8;17(2):216–32. doi: 10.1074/mcp.RA117.000470 (PMC5795388; doi:10.1074/mcp.RA117.000470)
Supplement: Supplemental Data [file supp_RA117.000470_133922_0_supp_23978_fzffwf.zip › spectra_annotation/mito_spectra_annotation/55-1-8-1-15-1.pdf]

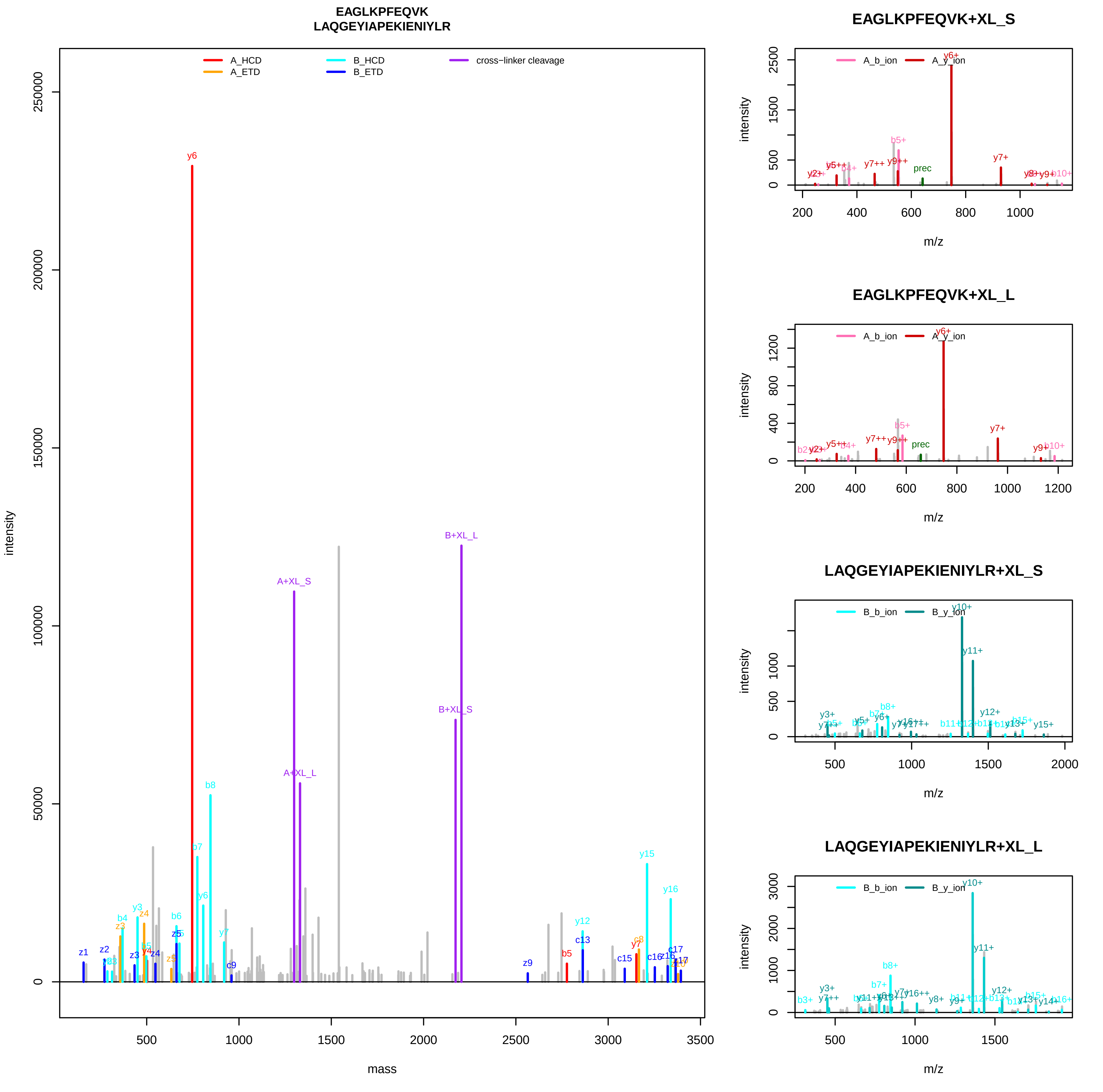

EAGLKPFEQVK
LAQGEYIAPEKIENIYLR
A_HCD
A_ETD
B_HCD
B_ETD
cross−linker cleavage
intensity
mass
0
50000
100000
150000
200000
250000
500
1000
1500
2000
2500
3000
3500
y6
A+XL_S
A+XL_L
B+XL_L
B+XL_S
b8
b7
y6
y7
b6
y3
b4
z3
z4
z1
z2
z3
z4
z5
c9
z9
b5
c13
y12
c15
y15
y16
c16
c17
EAGLKPFEQVK+XL_S
A_b_ion
A_y_ion
intensity
m/z
prec
y6+
y7+
b5+
EAGLKPFEQVK+XL_L
LAQGEYIAPEKIENIYLR+XL_S
B_b_ion
B_y_ion
y10+
y11+
y12+
LAQGEYIAPEKIENIYLR+XL_L
b8+
